# Low-threshold Sheet Optical Parametric Oscillator by Triply-resonant Cavity Phase Matching

**DOI:** 10.1038/s41598-019-55575-z

**Published:** 2019-12-17

**Authors:** Xing Wei, Xinjie Lv, ShiNing Zhu

**Affiliations:** 1 National Laboratory of Solid State Microstructures, School of Physics, and Collaborative Innovation Center of Advanced Microstructures, Nanjing University, Nanjing, 210093 China; 2 College of Engineering and Applied Sciences, Nanjing University, Nanjing, 210093 China

**Keywords:** Optics and photonics, Lasers, LEDs and light sources, Semiconductor lasers

## Abstract

Microcavity laser represents a novel type of integrated platform potentially allowing low-threshold and high-efficiency lasing behavior. Phase matching is a key parameter to achieve efficient lasing processes. Here we report a study on a triply-resonant phase-matching process in a sheet optical parametric oscillator. The oscillator contains an *x*-cut KTiOPO_4_ crystal, and the triply-resonant phase-matching was achieved by temperature tuning of the cavity. The measured oscillation threshold is as low as 13 *μJ* at 243.6 °*C* temperature along with a slope efficiency of 17.5% at 65.3 °*C*. Experimental results show that under some temperatures, triply-resonant sheet optical parametric oscillator can realize a limited number of longitudinal modes (n ≤ 3). Our results represent the first observation of triply-resonant phase-matching process in an on-chip optical parametric oscillator, opening up the possibilities on future applications including quantum chips, vertical-cavity surface-emitting lasers and narrow-band two-photon sources.

## Introduction

In recent years, optical microcavities have been widely applied to realize integrated chip-based devices and numerous applications in active and passive devices have been demonstrated^1–9^. Due to their high-Q values, flat microcavities (i.e. Fabry-Perot microcavities) can produce a unique spectrum and are commonly used to make compact laser sources^10,11^. In microcavity lasers consisting of nonlinear crystals, laser light of different wavelengths coexist in the cavity introducing phase mismatches in the parametric conversion process. Proper phase matching scheme is therefore a key to realize efficient lasing. To this end, in 1962 Bloembergen *et al*. proposed two approaches, namely quasi-phase-matching (QPM) and cavity phase matching (CPM)^12^. QPM compensates the phase mismatch by periodically reversing the polarization of the crystal with the period shorter than the coherent length. In the scheme of CPM, the crystal acts as a Fabry-Perot microcavity (FPMC) with its cavity length comparable with the coherence length, introducing constructive interference of the forward and backward propagating beams reflected from the front and rear crystal surfaces. This mechanism can greatly extend the distance of nonlinear action and thus improve the conversion efficiency. In 2011, Xie realized the concept of CPM in an on-chip optical parametric oscillator (OPO) and observed a single-longitudinal mode and narrow linewidth parametric output^13^. Till now several crystal materials have been used to realize CPM process, including LiNbO_3_ (LN), KTiOPO_4_ (KTP) and other non-ferroelectric crystal materials such as BBO and LBO crystals^14,15^. The conversion efficiency and thus the oscillation threshold of the FPMC is however limited by the crystal nonlinear coefficients, thickness as well as its fabrication accuracy.

In this work, we demonstrate a significant improvement of the CPM efficiency by using a sheet optical parametric oscillator (SOPO) via a triply-resonant phase-matching process. The SOPO consists of a dielectric nonlinear KTP crystal sheet, the two end faces of which were high-reflectance coated at the wavelength of pump, signal and idler beams, forming a high-Q FPMC. The pump wave was tuned on resonance with the cavity by varying the cavity temperature. Although the thickness of the sheet KTP crystal is less than one coherence length, the cavity effect strongly extends the nonlinear interaction length, exhibiting a high slope efficiency and high energy output of the converted signal and idler waves with a near-transform-limited spectral and near-diffraction-limited spatial features.

## Results and Discussion

### Theoretical analysis

In previously reported experiments, the parametric process only occurs in the forward direction with a single pass of the pump beam. In our scheme, pump continuously converts into parametric signal and idler light in both forward and backward directions. When the two directional parametric processes are in phase, constructive interference induces an enhancement of conversion efficiency. According to the coupled wave equation, under the slow-varying amplitude approximation, the phase mismatch $\epsilon$ after the reflection can be expressed as

1 $$\epsilon =\Delta k\cdot L-\Delta \varphi $$

where

2 $$\Delta \varphi ={\varphi }_{p}-{\varphi }_{s}-{\varphi }_{i},$$

**Figure 1:**
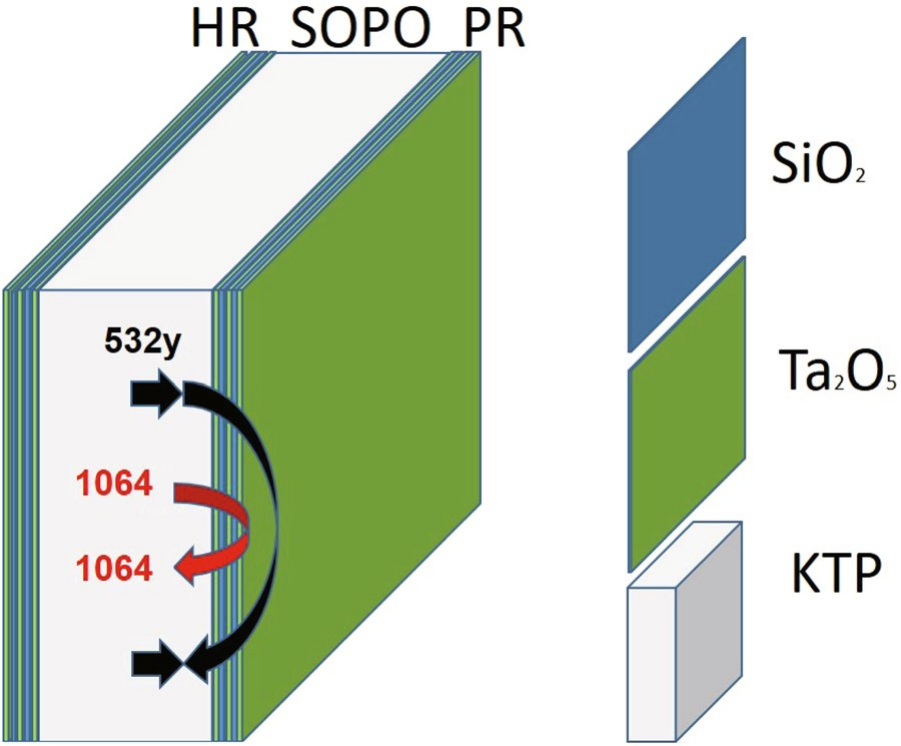
Structure of sheet optical parametric oscillator. The rear and front surface is 24- and 28-layer structure respectively.

**Figure 2:**
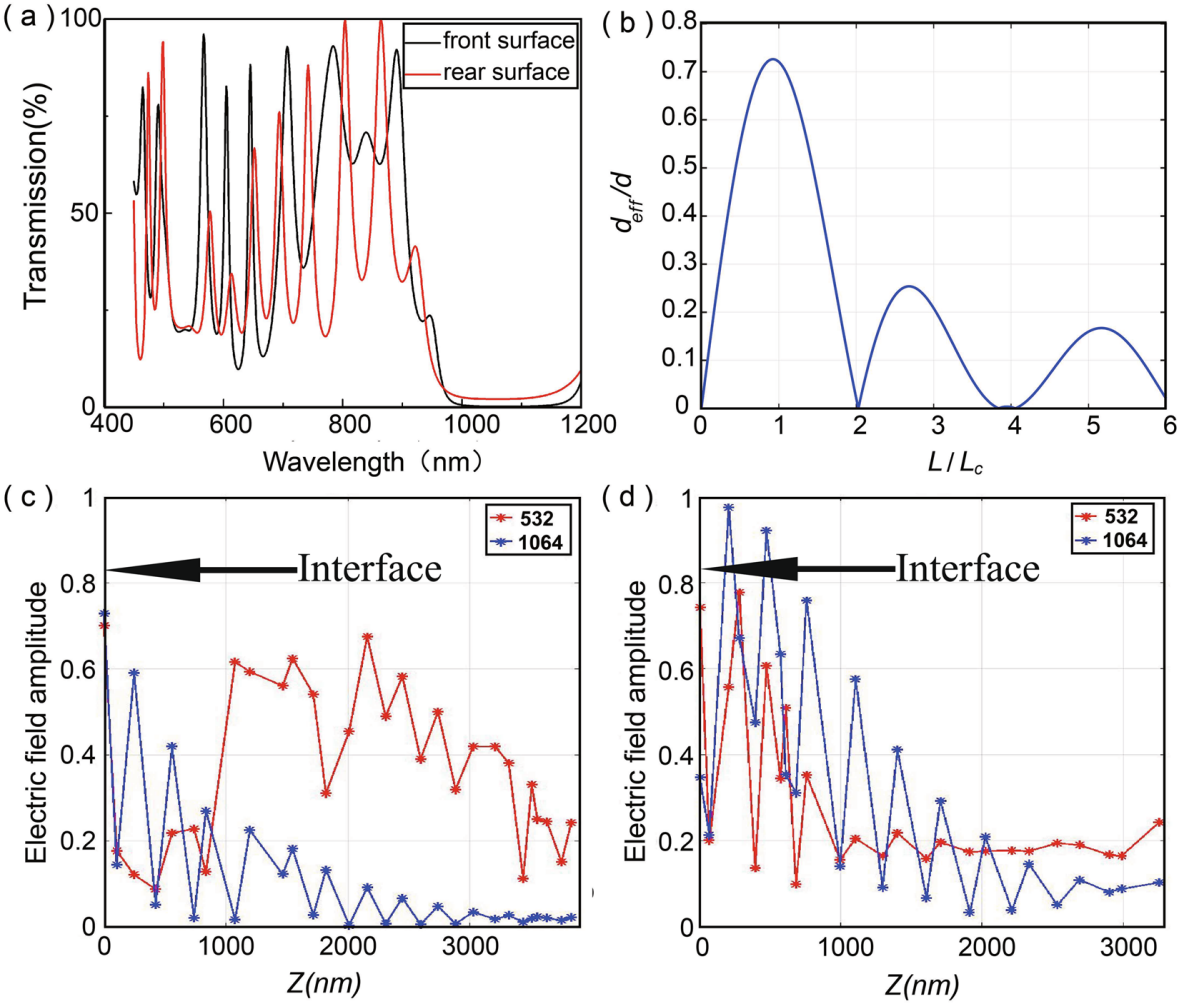
Simulated result: (**a**) Simulated transmissivity of SOPO as a function of wavelength. Black: front surface; red: rear surface. (**b**) Simulated d_*eff*_ ≤ d as a function of L ≤ L_*c*_. (**c**) Electric field amplitude at 532 nm (red) and 1064 nm (blue) wavelength as a function of propagation distance *z* in the front and (**d**) rear surface. The arrows in (**c**) and (**d**) mark the interface between KTP and SiO_2_. The electric field amplitude is normalized.

*φ*_*p*_, *φ*_*s*_, *φ*_*i*_ representing the phase shifts of the pump, signal, and idler waves at the crystal surfaces respectively,

3 $$\Delta k={k}_{p}-{k}_{s}-{k}_{i}$$

is the wavevector of the phase mismatch. Here k_*p*_, k_*s*_, k_*i*_ the wavevector of the pump, signal, and idler beams respectively. *L* is the cavity length. To precisely control the phase shift of all the beams at the crystal front and rear surfaces, we designed an alternate periodic structure as a metal-like dielectric coating (Fig. 1). The rear and front surface is a 24 and 28 layers of multilayer structure respectively. The red (black) curve in Fig. 2(a) plots the simulated transmission for rear surfaces, we designed an alternate periodic SiO_2_ and Ta_2_O_5_ as a metal-like dielectric coating (Fig. 1). The rear and front surface is a 24 and 28 layers of multilayer structure respectively. The red (black) curve in Fig. 2(a) plots the simulated transmission for the front (rear) surface as a function of wavelength. The black curve shows the transmission is 20% at 532 nm and is close to 0.2% at 1064 nm wavelength for the front surface. Similarly, for the rear surface, the red curve reveals a transmission of 20% at 532 nm and as low as 0.2% at 1064 nm. Taking Δ*φ* into consideration, the effective nonlinear coefficient d_*eff*_ is introduced as^16^:

4 $${d}_{eff}=d|sinc(\frac{\pi L}{2{L}_{c}})sin(\frac{\pi L}{2{L}_{c}})|$$

where *d* is the material nonlinear coefficient and *L*_*c*_ is the coherence length. Figure 2(b) plots *d*_*eff*_/*d* as a function of *L*/*L*_*c*_. It can be seen that the nonlinear coefficient varies with the cavity length. According to the simulation, each reflection the surface coating introduces a phase shift of 0.6*π* at wavelength close to 532 nm and 1064 nm, leading to a significant drop of conversion efficiency due to phase mismatch. When $\epsilon$ = 2*nπ*, the waves before and after the reflection surface are in phase giving a constructive interference. Figure 2(c,d) coordinate *x* = 0 mark the interface between the multilayer film and the crystal. Figure 2(c,d) plots respectively the numerically simulated electric field amplitude in the front and rear multilayer structures at 532 nm and 1064 nm wavelength using the transfer matrix of each layer. The electric field amplitude has been normalized with the maximum value. The position at *z* = 0 and 3830.98 nm represents the interface between KTP and SiO_2_. It can be seen that the field amplitude oscillates with the overall intensity decays over the propagation distance *z*. The observed intensity peaks at specific locations are originated from the multiple reflection and transmission induced coherent superposition.

The triple-resonance phase-matching condition is therefore given by: (1) $\epsilon$ = 2*nπ*, (2) longitudinal mode matching *ω*_*p*_ = *ω*_*s*_ + *ω*_*i*_. In our design, the surfaces film was designed to achieve $\epsilon$ = 2*nπ*, and we kept *L* ≤ *L*_*c*_ to realize efficient CPM. Specifically, result in Fig. 2(b) indicates that the most efficient process occurs where *L* ≤ 0.72 *L*_*c*_. The transmittance of FPMC reads as

5 $$T=\frac{1}{1+\frac{4R}{{(1-R)}^{2}}{\sin }^{2}(\frac{\varphi }{2})},$$

where

6 $$\varphi =\frac{4\pi nL}{\lambda },$$

and *λ* is the vacuum wavelength. To achieve cavity temperature tuning, the refractive index of KTP crystal was modelled by the Sellmeier equation:

7 $${n}^{2}={A}_{i}+\frac{{B}_{i}}{{\lambda }^{2}-{C}_{i}}-{D}_{i}{\lambda }^{2},\,(i=x,y,z)$$

with the temperature correction term given by

8 $$\Delta n(\lambda ,t)={n}_{1}(\lambda )(t-25\,^\circ C)+{n}_{2}(\lambda ){(t-25^\circ C)}^{2}.$$

From this equation, we can calculate the temperature dependence of the effective nonlinear coefficient of the SOPO.

## Experiment Results

**Figure 3:**
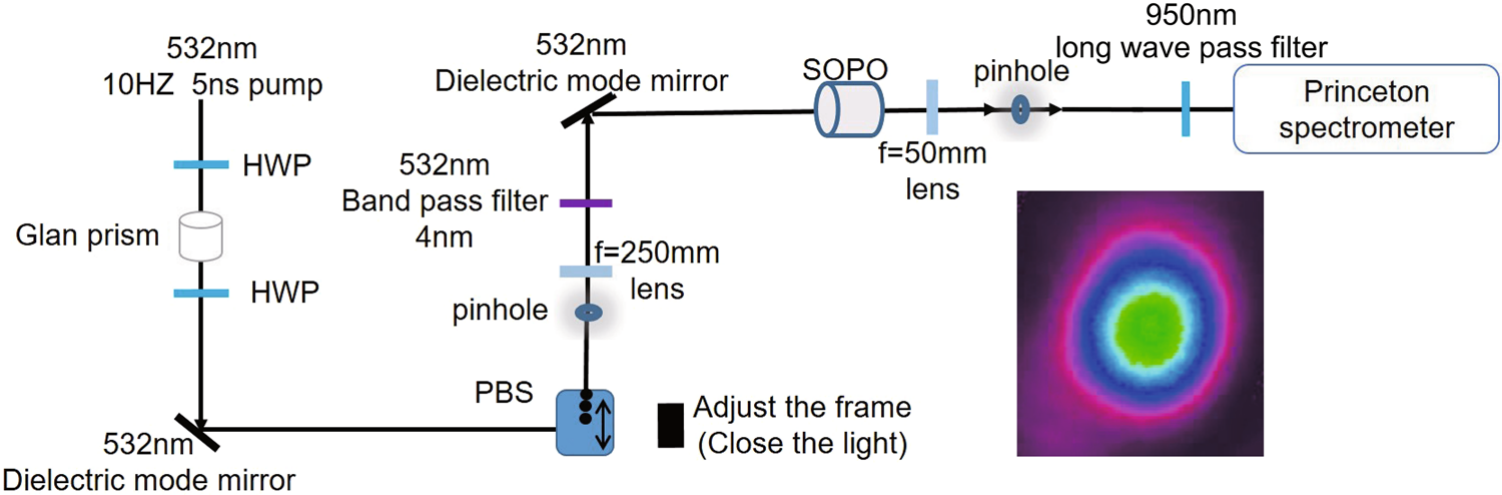
Sketch of experimental setup. HWP, half-wave plate; QWP, quarter-wave plate. Inset: one of the three longitudinal modes measured output profile using a CCD camera.

The experimental setup is described by Fig. 3. The SOPO was pumped by a single-longitudinal-mode frequency-double yttrium-aluminum-garnet laser at 532 nm wavelength. It was used as the pump source of a tunable OPO system (Sunlite, Continuum, Santa Clara, CA) with pulse duration of 5 ns and repetition rate of 10 Hz. The pump intensity can be adjusted by a half-wave plate (HWP) and a GLAN prism. The pump beam was firstly focused onto a small pinhole for spatial filtering (TEM_00_ mode), and then coupled into the SOPO. The SOPO was set in a temperature-controllable oven with an temperature tuning accuracy of 0.01 °*C*. The triply-resonance configuration requires a simultaneous longitudinal mode matching for signal and idler waves. With the proper setting of temperature, the signal and idler beams can be generated in pairs at TEM_00_ mode (see one of the three longitudinal modes measured intensity profile in the inset), will all the beams resonant in the cavity. The free-spectral range of the cavity is 172.5 GHz for 1064 nm wavelength, which is 168.62 GHz for 532 nm pump light. At the detection end, the pump wave was filtered by applying a long wave pass filter at 950 nm wavelength. The applied SOPO consists of *x*-cut KTP crystal sheets with the dimensions of 5 mm(*y*-axis) × 5 mm(*z*-axis) × 500 *μm*(*x*-axis). The pump and idle beams were set as the same polarization state along the *y*-axes, with the polarization of signal beam along the *z*-axes. The input surface of KTP crystal was coated as R = 80% for 532 nm, and R_1_ = 99.8% for 1064 nm wavelength. The output surface was coated as R = 80% for 532 nm and R_2_ = 98.0% for 1064 nm wavelength.

**Figure 4:**
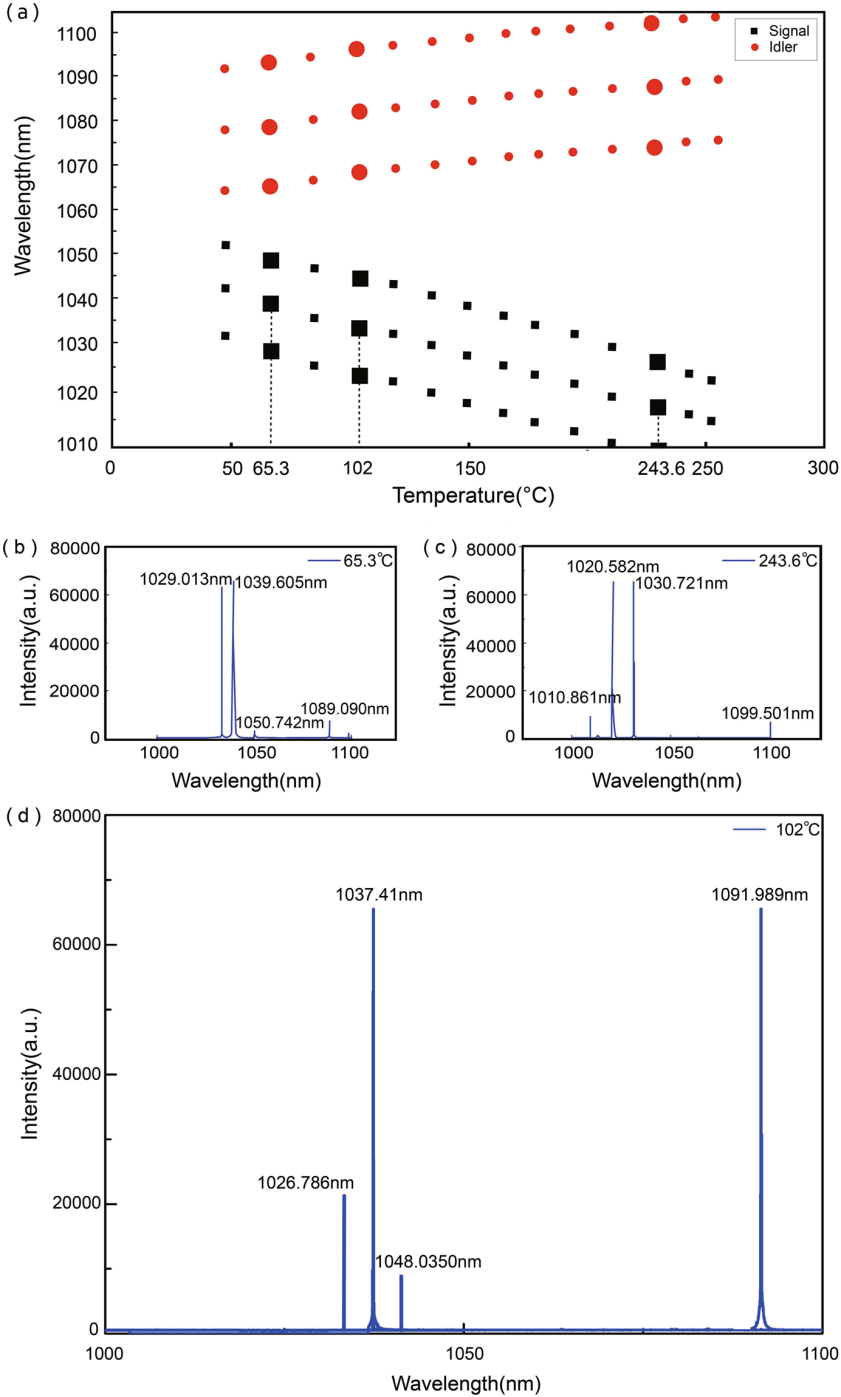
(**a**) Theoretically calculated output wavelength of signal(black squares) and idler waves (red dots) as a function of temperature. The heavier red and black dots represents the optimal phase matching points. (**b**) Measured spectra of signal and idler beams at *t* = 64.3 °*C* and (**c**) *t* = 243.6 °*C* respectively. The points with larger marker size correspond to data points in (**b**) and (**c**). (**d**) Measured spectra of signal and idler beams at *t* = 102 °*C*.

Figure 4(a) plots the theoretical calculated wavelength of the signal and idler wavelength as a function of temperature using Eqs. (5–8). It can be seen that the signal wavelength can be tuned within a range of 43.3 nm when the temperature varied from 65.3 °*C* to 243.6 °*C*. Optical parametric oscillation only works at discrete temperature points under which conditions the phase matching condition is fulfilled. We observed the cavity transmittance is almost unity when pump beam is resonant. At each resonant temperature (listed in Fig. 4(a)), there are three cavity modes fulfilling the phase matching condition, spaced with each other by 10 nm. The data points with larger marker size in Fig. 4(a) correspond to the temperatures in Fig. 4(b–d), showing the measured optical spectra at those temperatures. As the temperature changes, the intensity of the longitudinal mode will shift. The marked peaks in the Fig. 4(b–d) correspond to the signal (shorter wavelength) and idler waves (longer wavelength), with the measured wavelength agreeing excellently with the theory. Note that some of the wavelengths is not visible from the measurement due to the range limit of used spectrometer. Some of the tiny peaks were originated from the noise of the spectrometer.

**Figure 5:**
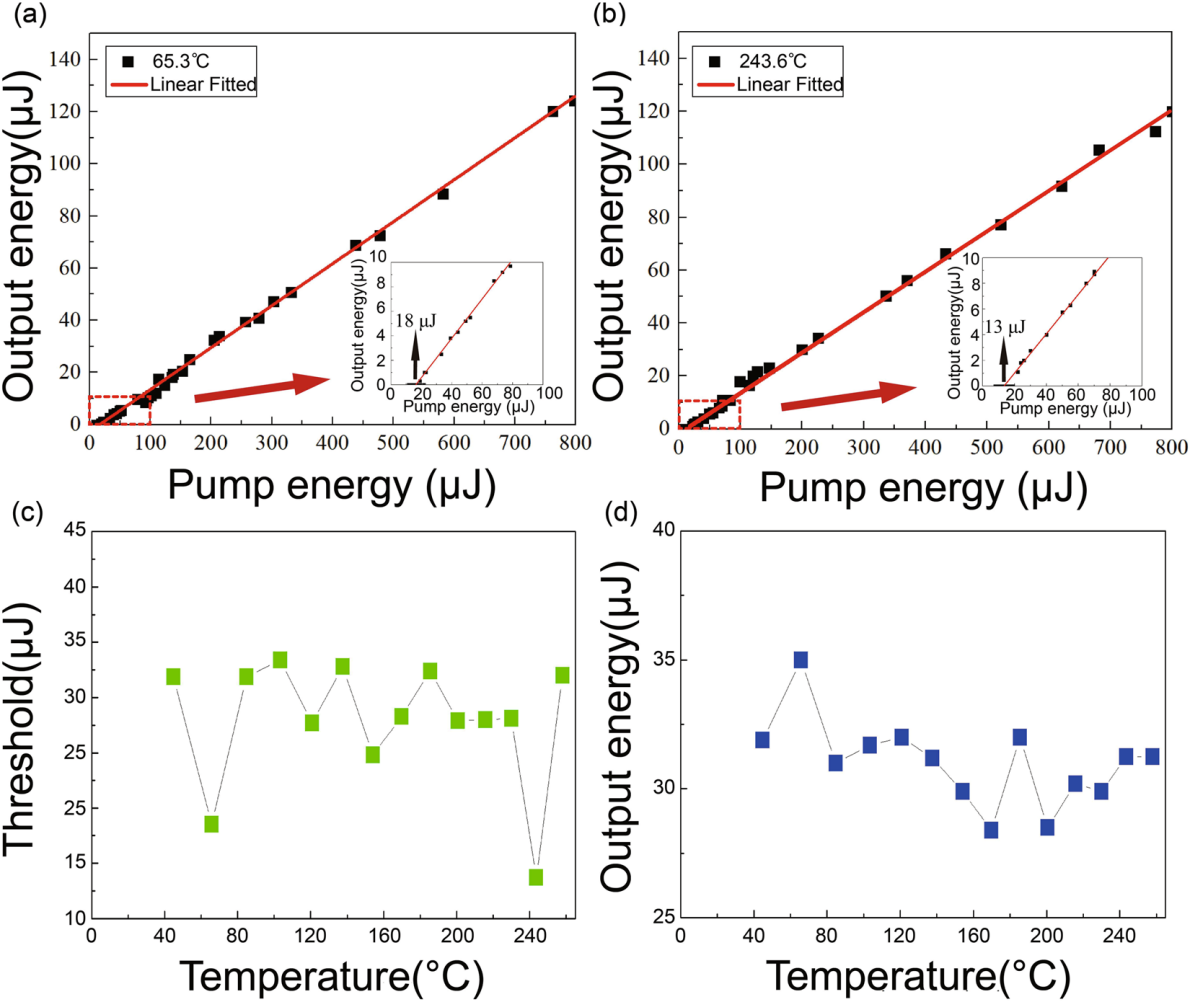
Measured output energy (containing signal and idler beams) as a function of input pump energy at *t* = 65.3 °*C* (**a**) and 243.6 °*C* (**b**). Inset: local enlargement near the oscillation threshold at 65.3 °*C* and 243.6 °*C*. The red lines represent the linear fitting. (**c**) Measured oscillation threshold and (**d**) Output energy of signal and idler waves as a function of temperature.

Figure 5(a) plots the measured output energy of signal and idler waves versus input pump energy at 63.6 °*C*, showing an oscillation threshold of 18 *μJ*, with a peak conversion efficiency of 18% (slope efficiency of 17.5% from the linear fit). Note the input power varies within 800 *μJ* to prevent potential damage of the sample. At 243.6 °*C* (Fig. 5(b)) the measured slope efficiency was 13.2%. The inset picture zooms the power range close to the oscillation threshold at 65.3 °*C* and 243.6 °*C*. Figure 5(c,d) displays respectively the measured oscillation threshold power and output energy (containing signal and idler waves) as a function of temperature. For the measurement in Fig. 5(d), the input energy is 225 *μJ*. The achieved lowest oscillation threshold value was 13 *μJ* at 243.6 °*C*, with the output energy remaining almost constant with temperature. As a comparison, the lowest threshold measured for the doubly resonant with single and double pump pass configuration (using the previously developed system) is 70 *μJ* and 30 *μJ* respectively, both of which is substantially higher than the triply resonant case reported in this work (13 *μJ*). Our temperature control furnace has a temperature control accuracy of 0.01 °*C* for the SOPO, which is relatively stable. The oscillation threshold formula of double resonance as follows^17^:

9 $$I=\frac{{\epsilon }_{0}{n}_{1}{n}_{2}{n}_{3}}{8{d}^{2}{\omega }_{s}{\omega }_{i}{L}^{2}}(1-{R}_{1})(1-{R}_{2}).$$

Here, ${\epsilon }_{0}$ is permittivity of vacuum, n_1_ = 1.7779, n_2_ = n_3_ = 1.7379. According to the oscillation threshold formula of double resonance, the intracavity resonance enhancement effect of pump light, and the reflectance of the front and rear end faces of pump is 80%, deriving the theoretical threshold outside the cavity is 2.5818 *μJ*. The cavity enhancement factor is given by Eq. (10):

10 $$\begin{array}{rcl}{A}_{circ} & = & {I}_{circ}/{I}_{laun}\\  & = & (|{E}_{circ}{|}^{2})/(|{E}_{laun}{|}^{2})\\  & = & 1/(|1-{R}_{1}{R}_{2}{e}^{(-i2\varphi )}{|}^{2})\\  & = & 1/({(1-\sqrt{({R}_{1}{R}_{2})})}^{2}+4\sqrt{({R}_{1}{R}_{2})}si{n}^{2}(\varphi )).\end{array}$$

where I_*circ*_ is the energy in the cavity, I_*laun*_ is pump light energy incident into the cavity, R_1_ = 80%, R_2_ = 80%, *φ* = 0, giving A_*circ*_ = 25. Since the pump transmission is 20%, the overall cavity enhancement factor is 5. The threshold of triple resonance is therefore 5 times of the double resonance, showing good agreement with experiment.

## Conclusion

In conclusion, we demonstrated a triply-resonant CPM with a oscillation threshold as lower as 13 *μJ* along with a slope efficiency of 17.5% in a SOPO system at 243.6 °*C*. The demonstrated single-longitudinal-mode and TEM_00_ mode output pave the way towards a high spectral and spatial brightness non-classical light for applications including high-fidelity quantum communication, state squeezing and quantum entanglement. The triply-resonant SOPO system can also be applied in the realizing miniaturized laser devices and integrated quantum chips.

## **Appendix**: thickness of each coated layer in FPMC

**Table:**

Layer	Material	Front face (nm)	Behind face (nm)
1	Ta_2_*O*_5_	80.56	65.65
2	SiO_2_	119.68	138.13
3	Ta_2_*O*_5_	78.72	74.36
4	SiO_2_	45.33	112.96
5	Ta_2_*O*_5_	70.49	80.1
6	SiO_2_	118.25	99.15
7	Ta_2_*O*_5_	111.98	37.58
8	SiO_2_	179.7	72.88
9	Ta_2_*O*_5_	144.1	74.34
10	SiO_2_	144.56	238.02
11	Ta_2_*O*_5_	142.19	110.15
12	SiO_2_	149.25	191.36
13	Ta_2_*O*_5_	137.36	104.91
14	SiO_2_	151.34	206.28
15	Ta_2_*O*_5_	147.81	98.5
16	SiO_2_	190.04	209.59
17	Ta_2_*O*_5_	103.83	108.79
18	SiO_2_	169.64	189.06
19	Ta_2_*O*_5_	81.77	118.38
20	SiO_2_	268.71	203.17
21	Ta_2_*O*_5_	120.5	156.34
22	SiO_2_	239.44	212.02
23	Ta_2_*O*_5_	97.92	86.08
24	SiO_2_	180.49	268.63
25	Ta_2_*O*_5_	133.52	
26	SiO_2_	176.91	
27	Ta_2_*O*_5_	143.33	
28	SiO_2_	103.56	

## References

[1] Foreman MR, Swaim JD, Vollmer F (2015). Whispering gallery mode sensors. Adv. Opt. Photon..

[2] Pöllinger M, ÓShea D, Warken F, Rauschenbeutel A (2009). Ultrahigh-Q tunable whispering-gallery-mode microresonator. Phys. Rev. Lett..

[3] Hoffmann M, Kopka P, Voges E (1997). Low-loss fibermatched low temperature PECVD waveguides with small-core dimensions for optical communication systems. IEEE Photon. Technol. Lett..

[4] Hoffmann M, Kopka P, Voges E (1998). Thermo-optical digital switch arrays in silica-on-silicon with defined zerovoltage state. J. Lightwave Technol..

[5] Pennetta R, Xie S, Russell PJ (2016). Tapered Glass-Fiber Microspike: High-Q Flexural Wave Resonator and Optically Driven Knudsen Pump. Phys. Rev. Lett..

[6] Xie S, Pennetta R, Russell PS (2016). Self-alignment of glass fiber nanospike by optomechanical back-action in hollowcore photonic crystal fiber. Optica.

[7] Gleine W, Muller J (1991). Laser trimming of SiON components for integrated optics. J. Lightwave Technol..

[8] Offrein B. J. *et al*. High contrast and low loss SiON optical waveguides by PECVD. In *Proceedings of the IEEE/LEOS Symposium Benelux Chapter*, November, pp. 290–293 (1996).

[9] Svalgaard M, Kristensen M, Directly UV (1997). written silicaon-silicon planar waveguides with low loss. Electron. Lett..

[10] Zeltner R, Pennetta R, Xie S, Russell PS (2018). Flying particle microlaser and temperature sensor in hollow-core photonic crystal fiber. Opt. Lett..

[11] Xie S (2016). Coherent octave-spanning mid-infrared supercontinuum generated in As_2_S_3_-silica double-nanospike waveguide pumped by femtosecond Cr:ZnS laser. Opt. Express.

[12] Armstrong JA (1962). Interactions between light waves in a nonlinear dielectric. Physical Review.

[13] Xie ZD (2011). Cavity phase matching via an optical parametric oscillator consisting of a dielectric nonlinear crystal sheet. Phys. Rev. Lett..

[14] Alford WJ, Smith AV (2001). Wavelength variation of the second-order nonlinear coefficients of KNbO3, KTiOPO4, KTiOAsO4, LiNbO_3_, LiIO_3_, *β*-BaB_2_O_4_, KH_2_PO_4_, and LiB_3_O_5_ crystals: a test of Miller wavelength scaling. JOSA B.

[15] Akbari R, Major A (2013). Optical, spectral and phasematching properties of BIBO, BBO and LBO crystals for optical parametric oscillation in the visible and nearinfrared wavelength ranges. Laser Physics.

[16] Lin HB (2013). High-performance cavity-phase matching by pump reflection. Optics Letters.

[17] Shi X. Q., Gong M. W. *Nonlinear Optics*. (First ed. Shi X. Q.) 90–92 (2001).

